# Worsening Inequalities in Child Injury Deaths in the WHO European Region

**DOI:** 10.3390/ijerph14101128

**Published:** 2017-09-26

**Authors:** Dinesh Sethi, Emogene Aldridge, Ivo Rakovac, Akash Makhija

**Affiliations:** 1 WHO Regional Office for Europe, Division of Noncommunicable Diseases and Promoting Health through the Life-Course, DK-2100 Copenhagen, Denmark; emogene.aldridge@hotmail.com (E.A.); akashmakhija22@gmail.com (A.M.); 2 WHO Regional Office for Europe, Division of Noncommunicable Diseases and Promoting Health through the Life-Course, WHO European Office for the Prevention and Control of Noncommunicable Diseases (NCD Office), 125009 Moscow, Russia; rakovaci@who.int

**Keywords:** children, injury, inequality, Europe

## Abstract

This article compares the mortality data for injuries in children aged 0–14 years in the World Health Organization WHO European region as estimated by the WHO Global Health Estimates for 2000 and 2015. While the region has seen a decline in child mortality due to injuries over the years, inequality persists between the low- and middle-income countries and high-income countries in the region. The gap in child mortality due to unintentional injuries has widened over the years between these two socioeconomic regions, particularly in terms of road injuries. In contrast, mortality rate ratios due to intentional injuries have narrowed between 2000 and 2015. The low- and middle-income countries need to scale up their efforts in injury prevention by adopting stricter regulations and higher safety practices to narrow the East-West gap in unintentional injuries.

## 1. Introduction

Injuries, whether unintentional or intentional, are the leading cause of mortality in children and adolescents in the World Health Organisation (WHO) European region [1,2]. There is a strong socioeconomic gradient, and populations in low- and middle-income countries have a higher risk of injury-related mortality in comparison to those in high-income countries (HIC) [2]. Income inequality and low economic level negatively affect child safety, and 90% of all childhood deaths globally from unintentional injury have been reported in low- and middle-income countries [3]. The United Nations Convention on the Rights of the Child (UNCRC) emphasises the importance of safeguarding the rights of a child to safe environments and recognises children’s vulnerability to injuries, whether these are unintentional or due to violence [4]. An injury is defined as physical damage that results when the body is subjected to sudden or brief intolerable levels of energy, that exceeds physiologic tolerance, or as the damage that results when the body is deprived of a vital element (e.g., oxygen) [5]. For the purpose of this paper, unintentional injuries include road injuries, poisonings, falls, thermal injuries, drowning, and mechanical forces, whereas intentional injuries result from self-harm, interpersonal violence, collective violence, and legal intervention (see Table 1). Both unintentional and intentional injuries are thought to be preventable [1,2,3,6,7,8,9]. The World Health Assembly in May 2011 adopted a resolution that urges member states to prioritise child injury prevention in national policy [10]. The aim of this paper is to examine mortality from childhood injuries using the recently published Global Health Estimates 2015 and to determine whether inequalities between HIC and low- to middle-income countries in the WHO European region have changed between 2000 and 2015 [11].

## 2. Materials and Methods 

### 2.1. Data Source

The Global Health Estimates 2015 (GHE) produced estimates using comparable methodology for the burden of disease, injury, and populations for the years 2000 and 2015, allowing for robust comparisons [11]. The WHO European region spans 53 countries, from Western Europe to parts of Central Asia, all with varying political, social, economic, and physical environments. They can be classified as lower-middle, upper-middle or HIC, based on information on gross national income provided by the World Bank [12]. L (lower-middle- and upper-middle-income countries (LMIC) with a gross national income of US$ 1026–12,475 (World Bank Atlas Method classification) are: Albania, Armenia, Azerbaijan, Belarus, Bosnia and Herzegovina, Bulgaria, Georgia, Kazakhstan, Kyrgyzstan, Montenegro, the Republic of Moldova, Romania, the Russian Federation, Serbia, Tajikistan, the former Yugoslav Republic of Macedonia, Turkey, Turkmenistan, Ukraine and Uzbekistan. HIC with a gross national income greater than US$ 12,475 are: Andorra, Austria, Belgium, Croatia, Cyprus, Czechia, Denmark, Estonia, Finland, France, Germany, Greece, Hungary, Iceland, Ireland, Israel, Italy, Latvia, Lithuania, Luxembourg, Malta, Monaco, The Netherlands, Norway, Poland, Portugal, San Marino, Slovakia, Slovenia, Spain, Sweden, Switzerland, and the United Kingdom). For the purpose of this paper, data for LMIC were compared with HIC. 

This study used data regarding all injuries and its subcategories as classified by the International Statistical Classification of Diseases and Related Health Problems version 10 (ICD-10, WHO, Geneva, Switzerland): unintentional (road injury, poisoning, falls, fire, heat and hot substances, drowning, exposure to mechanical forces, and other unintentional injuries) and intentional (self-harm, interpersonal violence, collective violence, and legal intervention) (see Table 1). Other unintentional injuries incorporate a range of causes including accidental threats to breathing (suffocation, choking, strangulation), contact with venomous animals and plants, or complications of medical or surgical care. 

### 2.2. Statistical Analyses

Mortality rates and subsequent rate ratios were calculated for children aged 0–14 years to compare rates between HIC and LMIC in 2000 and 2015. The difference in rate ratios between 2000 and 2015 was tested using Poisson exact tests for rate ratio comparisons [13]. All statistical analyses were performed using R version 3.3.3 (R Foundation for Statistical Computing, Vienna, Austria). The analytic approach used has been described previously [14].

## 3. Results

In 2015, there were a total of 18,328 child injury deaths in the WHO European region, a fall of 47% from the 34,603 deaths in 2000. The majority (88.5%) of the injury deaths were due to unintentional causes. Progress has been varied between injury types, and the proportionate fall in deaths due to intentional injuries was greater, decreasing by 55%, compared to a 46% decrease in unintentional injuries. Decreases occurred for all injury mechanisms and the greatest overall decrease was seen in collective violence (−73%), poisonings (−62%), and interpersonal violence (−60%) (Table 1). In 2015 the three leading causes of injury death in the region were other unintentional injuries, drowning, and road crashes. 

Despite overall progress, inequality between LMIC and HIC countries persists, and the mortality rates for all injury mechanisms are higher in LMIC compared to HIC. Declines in mortality from all injuries between 2000 and 2015 were greater in HIC than in LMIC, resulting in an increase in rate ratio between LMIC and HIC from 4.75 (95% Confidence interval (CI) 4.62–4.89) in year 2000 to 6.21 (95% CI 5.95–6.49) in year 2015 (31% increase, *p* < 0.001, ratio 1.31) (Table 2). 

However, marked differences by injury mechanism were observed. Between 2000 and 2015, the rate ratio for unintentional injuries increased by 41% (*p* < 0.001), mostly driven by faster declines in mortality in HIC from road traffic injuries (130% higher, *p* < 0.001), exposures to mechanical forces (21% higher, *p* < 0.05), and other unintentional injuries (27% higher, *p* < 0.001). 

In terms of mortality due to intentional injuries, although there is still inequality between HIC and LMIC, this gap is being bridged over the years. The mortality rate ratio between the two economic sub-regions has reduced by 22% (*p* < 0.001) between 2000 and 2015, because the fall in LMIC was greater than the fall in HIC during this period. Statistically significant changes can also be observed in the rate ratios for self-harm and interpersonal violence, both of which decreased by 28% and 18% respectively, between 2000 and 2015.

## 4. Discussion

Despite an overall fall in injury rates in children over the past 15 years, this study has shown that there is a widening of mortality rate ratios for all injuries between HIC and LMIC. This is mainly driven by the relatively smaller decline in unintentional injury in LMIC. Safety improvements in HIC are occurring at a faster pace than those in LMIC, leading to this divergence, which emphasises the need for improved policies in LMIC to ensure safer physical and social environments for children. Previous reports have described this growing inequality due to a faster pace of change in injury trends in HIC and have emphasised the East-West divide [1,14,15,16,17]. Further divergence has also been described for the period from 2000 to 2011 using an older edition of GHE [14,18]. In 2015, the three leading causes of injury death in children aged less than 15 years were from other unintentional injuries, drowning, and road crashes. 

The burden of mortality falls disproportionately on LMIC, where mortality rates of all injuries were 6.2 times higher than HIC in 2015. While most HIC have reduced child injuries, in part through the implementation of good practices for child safety, LMIC have failed to give adequate priority to child injury prevention [1]. The literature also suggests that the higher burden of injuries in the LMIC of Eastern Europe may be due to poorly managed transition to market economies, worsening inequalities in wealth, higher unemployment rates, decreased social capital, increased alcohol availability, and poor regulatory and enforcement mechanisms [19,20,21]. 

The strengths of this study are that it used the GHE 2015 data, which uses comparable methodology for mortality due to injuries and populations and which allows for robust comparison [11]. Classification and comparison of countries in two economic sub-regions would allow for greater precision of comparing rates than smaller population samples. However, this analysis is not without its limitations: the data compares between only two points of time, 2000 and 2015, between two country income groupings, without undertaking country comparisons. Nevertheless, the analysis highlights the inequalities in child mortality due to injury within Europe. Analysis of data within countries has not been undertaken and needs to be studied in order to prioritise programmes and target populations, including at the community level. Injury deaths are recorded as using a supplementary classification of external causes (V-X codes for ICD 10) and the consistency of use, completeness, and reliability of these records may vary in different countries, as may completeness of population denominator data. The interpretation of the codes may vary as well [22]. The analysis only uses mortality data and does not reflect the inequalities in magnitude of less severe injuries. Inequalities in injuries by country income have also been described for non-fatal injuries [23].

The different mechanisms of injury are worth further examination. Much of the increase in rate ratio between HIC and LMIC between 2000 and 2015 in injuries in children is driven by unintentional injuries. Of these, the mortality rate ratio between HIC and LMIC due to road traffic injuries has increased by 2.3 times between 2000 and 2015. Unfortunately, the rapid motorisation seen with economic development in LMIC has not been complemented by adequate development of legislative and regulatory mechanisms to improve vehicular safety and road infrastructure and modify road user risk behaviours [24,25]. Whereas most countries in the European region have reported a decline, many LMIC have reported an increase or levelling off of road traffic mortality rates [25]. Greater attention is being given to this area through a series of Global and European status reports on road safety [24,25] as well as the Decade of Action for Road Safety and the UN Road Safety Weeks that have advocated against speed and for pedestrian safety [26]. Greater attention also needs to be paid to inequalities within country, where those at socioeconomic disadvantage are more likely to be victims of road crashes [27,28]. Alcohol is a risk factor for road crashes, as well as other unintentional and intentional injuries. Alcohol use will influence risk behaviours and carer supervisory capacity [29]. The LMIC countries of Eastern Europe have the highest per head consumption of alcohol in the world [30], and the excessive burden of injuries from harmful alcohol use has been well documented [31].

Another leading category of death is other unintentional injuries, where the mortality rate ratio between HIC and LMIC has widened between 2000 and 2015. Though this grouping represents a broad range of injury mechanisms, some of these injuries occur in the home environment, which is a potential source of hazard as children spend a large part of their time at home [32]. LMIC have a larger proportion of unsafe housing stock with adverse living conditions associated with greater child injury mortality. Both country level income inequality and lower gross domestic product are associated with higher child injury mortality. Tackling housing conditions may help decrease the widening gap in child injuries in HIC and LMIC [33]. Many of the thermal injuries in children also occur in or around the home [1,5]. In 2015, thermal injury mortality rates were 9.8 times higher for LMIC than HIC. Previous analyses of paediatric mortality from burns showed that there was an inverse correlation with gross national income and income inequality in Eastern Europe and Central Asia, which is in keeping with the findings here of high burn mortality in LMIC [34].

Drowning is the third leading cause of death in children in the region, and the rates in LMIC are 9.2 times higher than in HIC. Exposure to expanses of unfenced areas of natural water, irrigation canals, and pools, as well as lack of safety equipment, life guards, training children in water safety skills, and supervision by carers are key areas that warrant further attention in LMIC [35]. Another reason that has been proposed for the greater improvement in mortality seen in HIC may be greater access to high quality trauma services for children, which this study was not able to consider [36]. 

In contrast to unintentional injuries, significant narrowing between the mortality rate ratios of HIC to LMIC have occurred for intentional injuries between 2010 and 2015. This is true for both interpersonal violence and self-inflicted injuries. Social disintegration, deregulation, and freer alcohol availability led to more intentional injuries from violence and suicide in Eastern Europe after the collapse of the former Soviet Union in the 1990s [37]. Since then, the proactive response of the LMIC governments to tackle violence and crime has led to the rapid fall in intentional injuries [38]. The success of these initiatives needs to be intensified and further studied. Investing in social protection will further safeguard children from interpersonal violence, and such benefits will be realised throughout the life course. Investing in social protection and preschool care have been emphasised as strategies for improving developmental outcomes in References [6,39,40,41]. 

This paper used the same analytical approach as described previously [9]. However, the results of this paper and the analysis described previously [9] cannot be compared directly, as the methods and data sources between each iteration of GHE are changing and the results cannot be compared directly [42]. Therefore, the analysis was repeated using the 2015 version of GHE and only the direction and magnitude of inequalities, which are mostly in line, were compared. 

## 5. Conclusions 

This study has shown that while there has been an overall decline in child injury mortality since 2000, inequalities in unintentional injury mortality between HIC and LMIC have widened. The results of the present study are mostly in line with the trends observed until 2011 [14], indicating no progress over the last four years in this regard. LMIC need to urgently scale up their efforts in child injury prevention in order to reduce the inequality gap. Otherwise, under the business as usual scenario, we will witness further widening of inequalities. LMIC can join the international calls to increase the policy priority to child injury prevention [1,3,10,43]. More recently, the Sustainable Development Goals (SDG) have included targets for injury and violence prevention: SDG 3.6 on road safety, 5.2 on violence against women and girls, SDG 11.2 on safe and sustainable infrastructure, and 16.2 on violence against children [44]. Further SDG goal 10 aims to reduce inequalities within and between countries. Investing in safe infrastructure and sustainable communities are critical to child injury prevention. The target on reducing child mortality (target 3.1) will not be achieved unless action is taken to reduce childhood injuries [44]. This provides an opportunity to harness intersectoral support from different actors and a whole of society approach as also emphasised by Health 2020 [45]. LMIC in the European region have made great strides in overcoming infectious disease mortality but now face the challenge of high injury mortality in children, as the relative importance of injuries has increased [16,46]. Though LMIC have been successful in bringing down childhood injury mortality rates over the years, they need to accelerate the progress, such as by adopting means used by HIC for child injury prevention [1,3]. More action is needed in LMIC by developing national policies and plans to coordinate the implementation of intersectoral programmes [47]. To respond to the challenge of injuries, LMIC need to invest in their leadership, infrastructure, and capacity [10,43,48]. Research suggests that a combination of legislative changes, vehicle and product design, engineering designs, and safe infrastructures and housing have been responsible for much of the successes, rather than focusing on individual behaviour change [16]. Injury prevention practitioners and policy makers should employ a whole of society approach and utilise the wide evidence base on prevention programming, to strengthen legislation and regulation, ultimately to develop safe environments and communities for children. An approach of societal accountability rather than individual responsibility is needed [1,38,46]. Reducing child injury inequities in Europe is a matter of social justice and is developmentally prudent. 

## Figures and Tables

**Table 1:** Percentage change the in number of deaths in children under 15 years of age by injury mechanism in 2000 and 2015 for WHO European region.

		2000		2015		2000–2015
Injury Mechanism	ICD-10 Codes	Deaths	Rate (Deaths per 100,000)	Deaths	Rate (Deaths per 100,000)	Difference in Deaths (%)
All injuries	V01–Y89 (minus X41–X42, X44, X45)	34,603	20.12	18,328	11.33	−47.0
Unintentional injuries	V01–X40, X43, X46–59, Y40–Y86, Y88, Y89	30,632	17.81	16,543	10.23	−46.0
Road injury	V01–V04, V06, V09–V80, V87, V89, V99	6411	3.73	3357	2.08	−47.6
Poisonings	X40, X43, X46–X48, X49	1752	1.02	666	0.41	−62.0
Falls	W00–W19	2480	1.44	1593	0.98	−35.8
Fire, heat, and hot substances	X00–X19	3273	1.90	1547	0.96	−52.7
Drowning	W65–W74	7949	4.62	3679	2.28	−53.7
Exposure to mechanical forces	W20–W38, W40–W43, W45, W46, W49–W52, W75, W76	2507	1.46	1694	1.05	−32.4
Natural disasters	X30–X39	73	0.04	13	0.01	−82.2
Other unintentional injuries	Rest of V, W39, W44, W53–W64, W77–W99, X20–X29, X50–X59, Y40–Y86, Y88, Y89	6186	3.60	3992	2.47	−35.5
Intentional injuries	X60–Y09, Y35–Y36, Y870, Y871	3971	2.31	1785	1.10	−55.0
Self-harm	X60–X84, Y870	1688	0.98	966	0.60	−42.8
Interpersonal violence	X85–Y09, Y871	1586	0.92	629	0.39	−60.3
Collective violence and legal intervention	Y35–Y36	698	0.41	191	0.12	−72.6

**Table 2:** Death rates in children aged 0–14 years in the WHO European region by injury mode in LMIC and HIC in years 2000 and 2015 and rate ratios between LMIC and HIC.

Injury Mechanism	Year 2000			Year 2015			Rate Ratio	2015:2000 Rate Ratio
	Mortality Rate per 100,000		Rate Ratio (95% CI)	Mortality Rate per 100,000		Rate Ratio (95% CI)	2015:2000	*p* Value
	LMIC	HIC	LMIC:HIC	LMIC	HIC	LMIC:HIC		
All injuries	32.18	6.77	4.75 (4.62–4.89)	19.15	3.08	6.21 (5.95–6.49)	1.31	<0.001
Unintentional injuries	28.58	5.89	4.85 (4.71–5.01)	17.49	2.56	6.82 (6.51–7.15)	1.41	<0.001
Road injury	4.74	2.61	1.82 (1.72–1.91)	3.29	0.79	4.18 (3.83–4.56)	2.30	<0.001
Poisonings	1.84	0.12	15.94 (12.94–19.83)	0.74	0.06	12.2 (9.09–16.73)	0.77	0.096
Falls	2.48	0.29	8.55 (7.48–9.82)	1.75	0.17	10.15 (8.51–12.2)	1.19	0.057
Fire, heat, and hot substances	3.27	0.39	8.49 (7.55–9.56)	1.70	0.17	9.75 (8.18–11.71)	1.15	0.126
Drowning	8.02	0.86	9.36 (8.66–10.13)	4.01	0.44	9.16 (8.2–10.26)	0.98	0.721
Exposure to mechanical forces	2.41	0.41	5.94 (5.29–6.69)	1.80	0.25	7.2 (6.2–8.4)	1.21	0.011
Natural disasters	0.05	0.03	1.63 (0.99–2.75)	0.02	0.00	N/A	N/A	0.005
Other unintentional injuries	5.77	1.19	4.84 (4.52–5.19)	4.17	0.68	6.15 (5.61–6.75)	1.27	<0.001
Intentional injuries	3.60	0.88	4.09 (3.77–4.44)	1.66	0.52	3.2 (2.86–3.58)	0.78	<0.001
Self-harm	1.61	0.28	5.67 (4.93–6.54)	0.94	0.23	4.08 (3.47–4.82)	0.72	<0.001
Interpersonal violence	1.22	0.59	2.07 (1.86–2.31)	0.49	0.29	1.69 (1.43–2)	0.82	0.017
Collective violence and legal intervention	0.77	0.01	125.24 (53.41–386.93)	0.23	0.00	180.07 (31.93–7149.51)	1.44	1.000
